# Wildland Fires Worsened Population Exposure to PM_2.5_ Pollution in the Contiguous United States

**DOI:** 10.1021/acs.est.3c05143

**Published:** 2023-11-09

**Authors:** Danlu Zhang, Wenhao Wang, Yuzhi Xi, Jianzhao Bi, Yun Hang, Qingyang Zhu, Qiang Pu, Howard Chang, Yang Liu

**Affiliations:** † Gangarosa Department of Environmental Health, Rollins School of Public Health, Emory University, Atlanta, Georgia 30322, United States; ‡ Department of Environmental and Occupational Health Sciences, School of Public Health, University of Washington, Seattle, Washington 98195, United States; § Department of Biostatistics and Bioinformatics, Rollins School of Public Health, Emory University, Atlanta, Georgia 30322, United States

**Keywords:** smoke PM_2.5_, wildfire, air pollution, remote sensing, machine learning

## Abstract

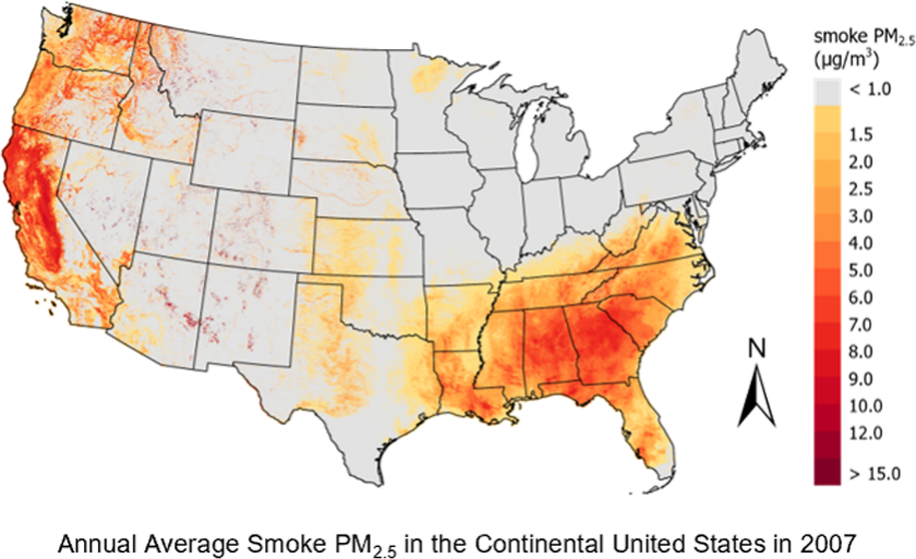

As wildland fires become more frequent and intense, fire smoke has significantly worsened the ambient air quality, posing greater health risks. To better understand the impact of wildfire smoke on air quality, we developed a modeling system to estimate daily PM_2.5_ concentrations attributed to both fire smoke and nonsmoke sources across the contiguous U.S. We found that wildfire smoke has the most significant impact on air quality in the West Coast, followed by the Southeastern U.S. Between 2007 and 2018, fire smoke contributed over 25% of daily PM_2.5_ concentrations at ∼40% of all regulatory air monitors in the EPA’s air quality system (AQS) for more than one month per year. People residing outside the vicinity of an EPA AQS monitor (defined by a 5 km radius) were subject to 36% more smoke impact days compared with those residing nearby. Lowering the national ambient air quality standard (NAAQS) for annual mean PM_2.5_ concentrations to between 9 and 10 μg/m^3^ would result in approximately 35–49% of the AQS monitors falling in nonattainment areas, taking into account the impact of fire smoke. If fire smoke contribution is excluded, this percentage would be reduced by 6 and 9%, demonstrating the significant negative impact of wildland fires on air quality.

## Abstract 2

Wildfire smoke significantly impacts the air quality of a vast majority of the US population and places a greater burden on approximately 70 million suburban and rural residents.

## Introduction

With a changing climate, large-scale wildfire events have increased in frequency and intensity, and fire seasons have been prolonged in the contiguous U.S. (CONUS) in recent decades.^1,2^ Wildfire smoke contains large quantities of fine particulate matter (PM_2.5_, airborne particles with diameters smaller than 2.5 μm) and can adversely affect regional air quality in downwind communities that are tens to hundreds of kilometers away. For instance, Jaffe et al. reported that PM_2.5_ levels have increased in summer due to wildland fires in the western U.S.,^3^ and Geng et. al observed an significant enhancement in PM_2.5_ concentrations in intensive wildfire years in Colorado.^4^ This impact has become so expansive that a previous analysis of PM_2.5_ measurements from U.S. EPA’s ground monitoring network between 1988 and 2016 attributed the increasing trend of 98th quantile of 24 h PM_2.5_ concentration in the Northwestern U.S., in contrast to the decreasing trend in the rest of the contiguous U.S., to the influence of wildland fires.^5^ In January 2023, the U.S. EPA proposed to revise the National Ambient Air Quality Standards (NAAQS) of PM_2.5_ by lowering the primary annual PM_2.5_ standard to a range of 9.0–10.0 μg/m.^3^^6^ Previous studies documented that starting from 2016 or earlier, the influence of wildfire smoke has shaped the trajectories of average annual PM_2.5_ levels in approximately 75% of contiguous U.S.,^7^ and attainment under the new annual PM_2.5_ standard will be more challenging in fire prone regions.

Different from ambient PM_2.5_, smoke PM_2.5_ contains 5–20% elemental carbon (EC) and at least 50% organic carbon (OC) including many polar organic compounds.^8^ The greater oxidative potential of smoke PM_2.5_ implies the possibility of greater toxicity than ambient PM_2.5_.^9^ Recently, new aircraft-based campaigns, including Western Wildfire Experiment for Cloud chemistry, Aerosol absorption, and Nitrogen (WE-CAN) and Fire Influence on Regional to Global Environments and Air Quality (FIREX-AQ), have provided more details of smoke PM_2.5_ components for specific wildland fires.^10,11^ In addition, with the expanding wildland–urban interface and an aging U.S. population, the overall burden of wildfire-related diseases is expected to increase.^12^ A few previous studies have linked exposure to wildfire smoke PM_2.5_ with a series of adverse health outcomes including cardiovascular, respiratory, and mental health diseases.^12−13,15^ For example, Alman et al. positively associated short-term exposure of PM_2.5_ from wildfire with respiratory illnesses.^16^ Stowell et al. reported significant association between smoke PM_2.5_ exposure and a greater risk of emergency department visits due to asthma attacks after controlling for PM_2.5_ exposure from nonsmoke sources in Colorado.^17^

While chronic exposure to ambient PM_2.5_ has been shown to present a much greater risk to human health than acute exposure,^18^ few studies have assessed the health effects of chronic wildfire smoke PM_2.5_ exposure primarily due to the challenge of estimating long-term wildfire smoke PM_2.5_ exposure at high spatial and temporal resolutions. Since most wildland fires started in remote areas, regulatory monitoring networks such as the US EPA’s Air Quality System (AQS) are often insufficient to characterize the spatial patterns of smoke PM_2.5_. Low-cost sensor networks have been rapidly evolving to become a valuable complement to regulatory monitoring systems, primarily because of their broad spatial coverage and high sampling frequencies.^19^ PurpleAir is a citizen-based real-time PM_2.5_ monitoring network with nearly 10,000 sensors currently online globally.^20^ By utilizing various adjustment techniques,^21,22^ previous studies suggested that the low-cost sensor can be an important supplement to the reference ground monitors in PM_2.5_ exposure assessments.^23,24^ For example, Vu et al, incorporated the PurpleAir network with AQS monitors in estimating regional PM_2.5_ levels during a fire event in California.^25^ In addition to the limited spatial pattern, ground observations alone cannot separate fire smoke PM_2.5_ from other sources. Chemical transport models (CTMs) such as the Community Multiscale Air Quality (CMAQ) model can simulate fire-specific PM_2.5_ with full coverage in space and time, greatly expanding the study population of air pollution epidemiological studies to cover both urban and rural populations.^4^ However, uncalibrated CTM smoke simulations frequently suffer from substantial prediction errors caused by imperfect characterization of complex fire chemistry, inaccurate emission inventory, and rapidly changing local meteorology surrounding fires.^26^ Most recently, machine learning or statistical models that integrated ground observations, satellite remote sensing data, land cover and land use information, as well as CTM simulations have shown great promise to generate long-term, accurate, and high-resolution ambient PM_2.5_ concentrations worldwide with full spatial and temporal coverage. To date, a handful of non-CTM-based fusion models to estimate smoke PM_2.5_ levels have been reported. For example, O′Dell et al. (2019) estimated the contribution of wildland fire smoke to seasonal mean PM_2.5_ levels in the CONUS at a spatial resolution of ∼15 km.^27^ Childs et al. (2020) estimated daily smoke PM_2.5_ concentrations at 10 km spatial resolution using satellite-based fire smoke contours to define fire days. The coarse spatial resolutions of these studies cannot capture the detailed spatial gradients of the smoke PM_2.5_ levels. The lack of ground observations near the fires to be included in model training can also be attributed to the underestimation of peak smoke PM_2.5_ concentrations in these studies.

Here, we designed a multistage, CTM-based modeling framework to estimate full coverage, daily smoke PM_2.5_ concentrations in the CONUS at 1 km spatial resolution. This framework integrated CMAQ PM_2.5_ simulations, multiple satellite remote sensing products, meteorology reanalysis, land cover and land use information, and ground observations from both regulatory and low-cost sensor networks. Taking advantage of the high spatial and temporal resolution of our model predictions, we investigated the long-term impact of wildland fires on national air quality as well as the representativeness of the AQS monitoring network in estimating population exposure to fire smoke. In addition, we investigated the impact of lowering the PM_2.5_ standard on the attainment areas and the number of individuals affected by it, both with and without the influence of smoke emissions from fires.

## Materials and Methods

### Ground PM_2.5_ Measurements and Calibrations

We obtained Environmental Protection Agency (EPA) federal reference and acceptable ground PM_2.5_ measurements which were publicly available at the AQS.^28^ We calculated daily PM_2.5_ concentrations by averaging the hourly measurements at stations and days with at least 16 of 24 possible measurements. The rapidly developing low-cost sensor networks are a significant supplement of traditional monitoring due to their high spatial density and temporal frequency.^29,30^ We included measurements from the PurpleAir low-cost PM_2.5_ sensors to extend the spatiotemporal coverage of ground monitoring and increase the probability of capturing the PM_2.5_ pollutions from wildfire smoke.^31^ Since the PurpleAir PM_2.5_ measurements have biases when compared with reference-grade measurements, we performed a series of quality control and adjustment.^20^ We first removed all station days with less than 16 hourly measurements and those with 30% relative difference among two channels, which are measurements from two independent laser counters in each PurpleAir unit. We also removed daily values with PM_2.5_ levels above 1000 μg/m^3^, temperature less than −20 F° or higher than 140 F°, and humidity less than 0% or higher than 100%. We conduct geographically weighted regression (GWR) to adjust PurpleAir measurements which is similar to many previous studies.^24^ In order to perform a spatially representative adjustment across the entire study domain, we matched PurpleAir monitors and AQS stations within 5 km. A total of 230 AQS stations were paired, approximately half of which are located in the western U.S. Since meteorological conditions such as relative humidity and temperature have great impacts on PurpleAir accuracy,^24^ we divided CONUS into 4 subregions, as shown in Figure S1. We developed four regional GWR models with relative humidity and temperature as model covariates to adjust the PurpleAir measurements. A 20 km buffer was created for each region, and adjusted PurpleAir observations located in the buffers were calculated as the mean of two GWR models’ outputs in order to make a smooth transition between regions. The overall *R*^2^ between measurements of PurpleAir and their matched AQS monitors is 0.92 after adjustment and *R*^2^ varied from 0.79 to 0.96 among four regions. Adjusted daily PurpleAir observations over the annual standard of 12 μg/m^3^ were added to our final model.

### CMAQ Simulations

The Community Multiscale Air Quality (CMAQ) model is an atmospheric chemical transport model that combines emission sources, weather-based atmospheric transport, dispersion, chemical transformation, and deposition to predict air pollution concentrations.^32^ In this study, two sets of CMAQ model runs were used to predict daily ground PM_2.5_ concentrations at ∼12 km spatial resolution. While the full model simulated the total PM_2.5_ concentrations using all emission sources, the nonfire model toggled off wildland fire emissions to predict the nonfire PM_2.5_ concentrations. Detailed descriptions of the CMAQ model are published elsewhere.^33,34^ The model versions, emissions, and model configuration information on each model year in our study are presented in Table S1. In terms of the fire emission inventories, the models incorporated the BlueSky framework (v3.5.1) to represent both wildland and prescribed burning. We calculated the smoke PM_2.5_ concentrations by subtracting nonfire PM_2.5_ concentrations from the total PM_2.5_. Additionally, we determined the ratio of smoke PM_2.5_ by dividing smoke PM_2.5_ by the total PM_2.5_.

### Data Integration

A large array of predictor variables was used to develop the PM_2.5_ models, including satellite-retrieved aerosol, cloud, and smoke plumes information, gridded meteorology, population, land cover, and topographic data (detailed descriptions provided in the Supporting Information).

All data sets at various spatial resolutions were integrated at the 1 km grid of the multi-angle implementation of atmospheric correction (MAIAC) aerosol optical depth (AOD). Due to the missing data issue in MAIAC AOD, we applied a two-step gap-filling approach to obtain a full-coverage MAIAC AOD (detailed descriptions provided in the Supporting Information). Daily average PM_2.5_ measurements from the AQS monitors and PurpleAir sensors were assigned to their collocated grid cells, and averaged PM_2.5_ measurements were calculated at grid cells with multiple monitors. Note that the PurpleAir data were adjusted based on a previously reported method before merging with AQS measurements.^24^ We interpolated the coarse resolution variables into 1 km resolution using inverse distance weighting.^35^ They include CMAQ, Copernicus Atmosphere Monitoring Service (CAMS) AOD and meteorological variables. We obtained the land cover data at 30 m resolution from the National Land Cover Database. We collected road network and elevation data from the Global Roads Inventory Project and the Global Digital Elevation Model, version 3, respectively. For each grid cell, we calculated the percentages of land cover types, average elevations, and total road length. We matched our grid with the 1 km resolution population density data, which is from the Landscan Program at Oak Ridge National Laboratory (ORNL).^36^ We calculated daily total smoke plumes duration, daily weighted average plume density for each grid cell using the fire smoke polygons produced by the National Oceanic and Atmospheric Administration (NOAA) Hazard Mapping System.^37,38^ Terra and Aqua Moderate Resolution Imaging Spectroradiometer (MODIS) cloud fractions at 5 km resolution were assigned to the overlapped grid cells and then averaged if available. One weakness lies in capturing the diurnal cycle of PM_2.5_, as fire smoke transport differs between day and night due to varying meteorological conditions, and it is challenging for satellite instruments to capture nighttime fire smoke.^39−41^ Given the challenges in distinguishing daytime from nighttime meteorology and the limited understanding of nighttime meteorological effects on fires, our primary focus is on daytime conditions. Consequently, we calculated daytime averages of meteorological data as daily averages. Based on climate types, CONUS is divided into nine climate regions,^42^ and indicators of climate region were assigned to the overlapped grid cells.

### Smoke PM_2.5_ Model Development

Random forest (RF) is an ensemble algorithm based on multiple decision trees, and the outputs from all decision trees are averaged to be the prediction of the dependent variable.^43,44^ Each decision tree is built on a bootstrap training data, and a subset of independent variables are randomly selected in each tree node.^43^ The bootstrap strategy allows RF to be a robust model against overfitting.^44^ RF also provides an estimated importance rank which informs the weights of predictors and allows an easier interpretation, comparing to neural network models.^43,45^ The *R*^2^ and root mean squared error (RMSE) were calculated from overall, spatial, and temporal 20-fold cross-validation (CV), and we used them to assess the model performance and furthermore adjust model parameters.

**Figure 1:**
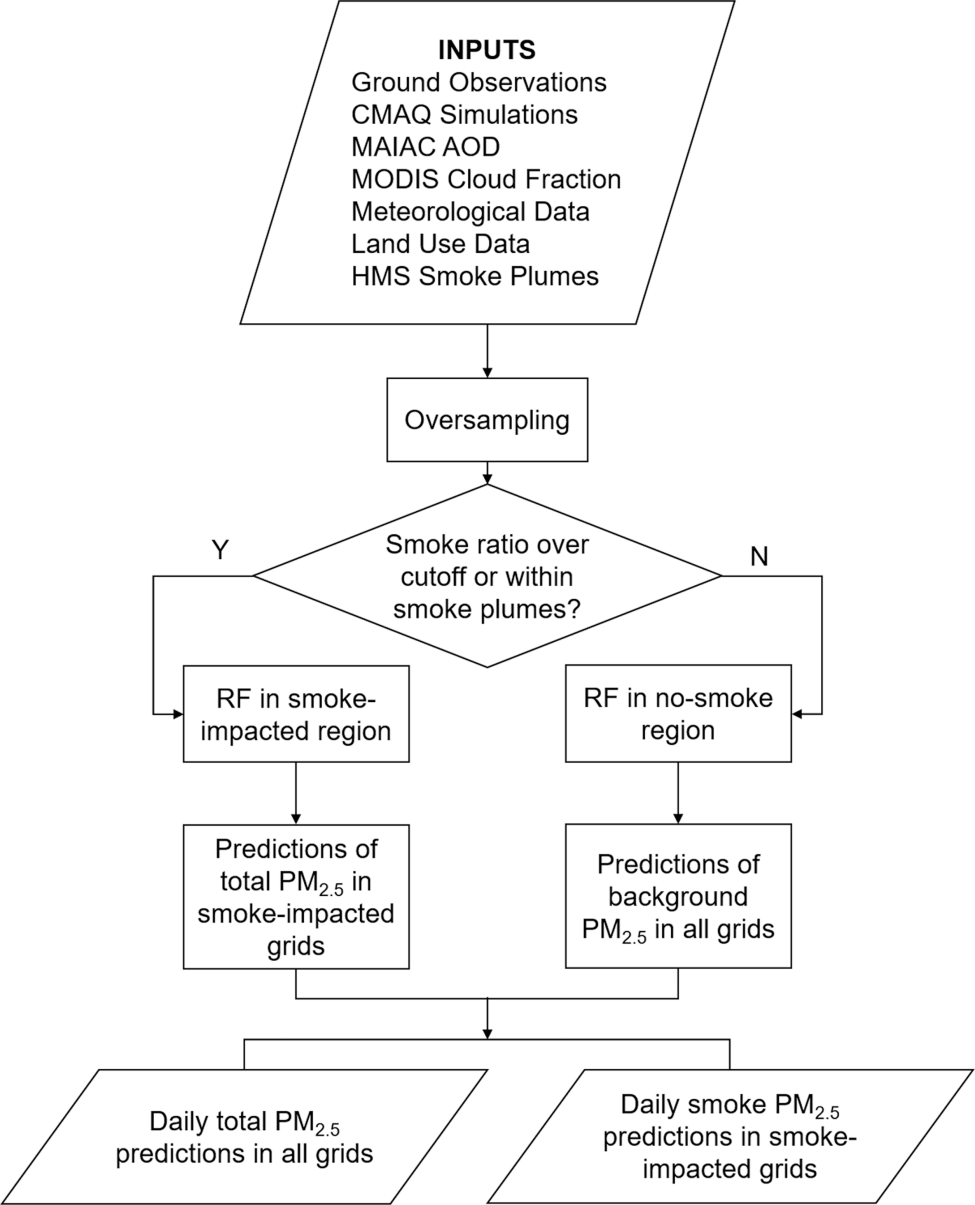
Flow diagram of the PM_2.5_ modeling framework (RF: Random Forest model).

Two random forest algorithms were trained independently in order to separate smoke PM_2.5_ from the background PM_2.5_ by grid cells and days (Figure 1). The smoke-impacted regions and no-smoke regions were defined by smoke impacts in both time and space. First, the modeling grid cells and days were divided into smoke-impacted regions and no-smoke regions according to daily HMS smoke plume polygons and the CMAQ smoke ratio (i.e., simulated smoke PM_2.5_ over the total PM_2.5_ concentration). On a given day, a smoke-impacted grid cell was defined as either being inside an HMS smoke plume polygon or having a CMAQ smoke ratio greater than a threshold. We examined different values of the CMAQ smoke ratio between 0.01 and 0.1, and training data sets were most balanced for two models with the ratio of 0.03. Next, in the smoke-impacted region, a random forest algorithm was trained to estimate daily total PM_2.5_ concentrations, which was assumed to be the sum of smoke contribution and background (i.e., contribution from all of the other sources). In the no-smoke region, smoke contribution was assumed to be negligible, and a separate random forest algorithm was trained to estimate daily background PM_2.5_ concentrations in the no-smoke region. Then, this no-smoke algorithm was also used to predict daily background PM_2.5_ concentrations in the smoke-impacted region. Finally, the daily smoke PM_2.5_ concentration in each grid cell of the smoke-impacted region was then calculated as the difference between the predicted total PM_2.5_ concentration and the predicted background PM_2.5_ concentration. Since only a small proportion of extreme-high ground PM_2.5_ concentrations were captured by AQS data, we applied a Synthetic Minority Oversampling Technique (SMOTE) to oversample the underrepresented measurements with high levels to improve the model performance at high PM_2.5_ concentrations.^25,46^ SMOTE generated synthetic samples along with their predictions from the five nearest grid cells in the training data set.^46^ PM_2.5_ concentrations over 35 (U.S. NAAQS for 24 h PM_2.5_) and below 100 μg/m^3^ were oversampled once, while the PM_2.5_ measurements over 100 μg/m^3^ were oversampled twice through SMOTE. The oversampled data accounted for 0.85% of the total input data, and the SMOTE process did not skew the distribution of PM_2.5_ observations. Our final training data set for smoke-impacted and no-smoke models had 1 681 873 and 2 010 266 station-day observations, respectively.

The formulas of models in smoke-impacted and no-smoke regions are
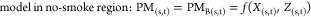

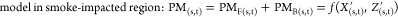
where PM_(s,t)_, PM_F(s,t)_, and PM_B(s,t)_ denote the ground-level PM_2.5_ concentration, fire component PM_2.5_ and nonfire background PM_2.5_ at location s on day t, respectively. For the model in no-smoke region, *X*_(s,t)_ is the CMAQ-simulated background PM_2.5_ at location s on day t, and *Z*_(s,t)_ is a vector of additional predictors, including gap-filled MAIAC AOD, meteorological factors, cloud fractions, land cover, and climate region types, as listed in Table S2. For the model in the smoke-impacted region, *X*′_(s,t)_ is the CMAQ-simulated total PM_2.5_ at location s on day t, while *Z*′_(s,t)_ includes the HMS data and all predictors in *Z*_(s,t)_.

## Results and Discussion

### Model Performance

Removing the oversampled data, the *R*^2^ of overall, spatial, and temporal CV of smoke-impacted model is 0.75 (RMSE = 4.59 μg/m^3^), 0.59 (RMSE = 5.88 μg/m^3^), and 0.67 (RMSE = 5.18 μg/m^3^), respectively, indicating a good model performance in fire grids. For the no-smoke model, the *R*^2^ of random, spatial, and temporal CV is 0.68, 0.47, and 0.63, with RMSE of 3.35, 4.30, and 3.59 μg/m^3^, respectively, which indicates the satisfactory performance from the random forest model for background PM_2.5_. As shown in Figure S2, when daily model estimations were compared with AQS measurements, random forest models slightly overestimated at low PM_2.5_ concentrations and underestimated at high PM_2.5_ values, especially when the daily PM_2.5_ concentration exceeds 100 μg/m^3^. After aggregating the daily PM_2.5_ predictions to a monthly level, the *R*^2^ of smoke-impacted and no-smoke models in the overall 20-fold CV increased to 0.84 and 0.78, respectively. This indicated that the majority of overestimations and underestimations are most likely randomly distributed, since random errors can be reduced by averaging.^47^ Scatter plots for aggregated monthly CV are shown in Figure S3. Same process was used for spatial and temporal CV and as a result, the *R*^2^ of both smoke-impacted and no-smoke models were improved, as shown in Table S3. After aggregating the overall CV to an annual level, the *R*^2^ between all predictions and AQS measurements is 0.9, implying a high accuracy of model predictions. As for variable importance, CMAQ is the most important predictor in both smoke-impacted and no-smoke models, and AOD and wind are the common parameters ranked in the top five in two models (Figure S4).

### Spatiotemporal Patterns of Smoke PM_2.5_ across the CONUS

**Figure 2:**
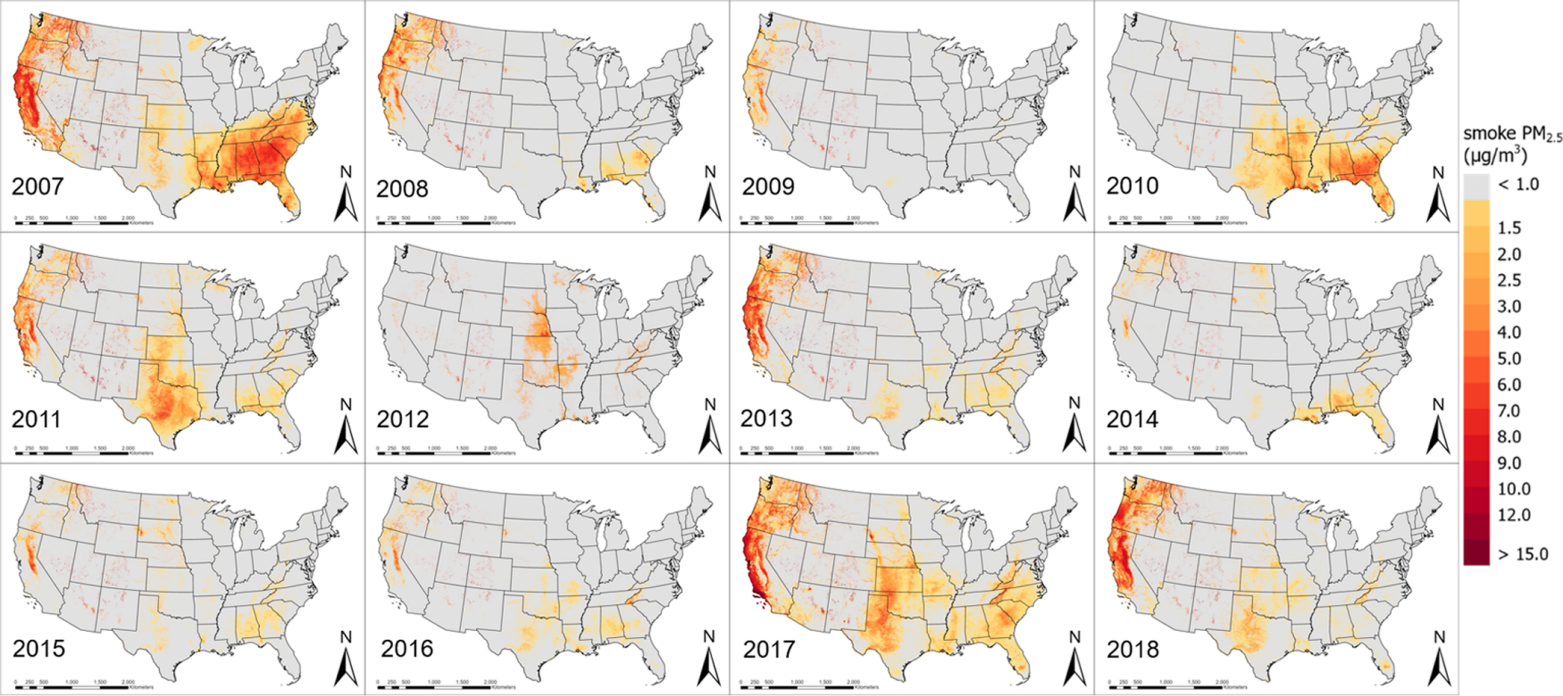
Annual mean smoke PM_2.5_ concentration (μg/m^3^) from 2007 to 2018 in the CONUS.

Figure 2 presents spatial distributions of annual mean smoke PM_2.5_ in the CONUS from 2007 to 2018, with a focus on identifying areas where annual average fire smoke exceeding 1 μg/m^3^ is deemed to have a significant impact on PM_2.5_ levels. While the Western U.S. has seen a significant and more persistent impact of fire smoke on PM_2.5_ levels, other regions including the mid-West and the Southeast have also suffered high smoke PM_2.5_ in certain years. For example, annual average smoke PM_2.5_ concentrations over 8 μg/m^3^ occurred in California, Oregon, and Washington in 2007–2009, 2011, 2013, 2017, and 2018, and over 50% of the areas in these states were impacted by fire smoke during these years. Along the California coasts and in the Central Valley, annual average smoke PM_2.5_ concentrations exceeded 12 μg/m^3^ in 2007, 2017, and 2018. We observed the highest annual average wildfire smoke PM_2.5_ level north of Ventura County in Southern California at 25 μg/m^3^ in 2017. Other Western states, such as Idaho, Montana, Utah, Colorado, Arizona, and New Mexico have been affected to a lesser degree, with annual mean smoke PM_2.5_ levels ranging between 0 and 5 μg/m^3^. The second most affected region by fire smoke is the Southeast. For example, annual smoke PM_2.5_ levels of up to 9 μg/m^3^ were common in Alabama, Georgia, and Carolinas. Fire smoke also contributed significantly to elevated PM_2.5_ levels in Georgia and Florida in 2010 and 2017. In addition, air quality in the Midwestern states was periodically affected by fire smoke. For example, approximately half of Texas, Oklahoma, and Kansas showed detectable fire smoke impact in 2010, 2011, and 2017, with high smoke PM_2.5_ levels observed over large cities such as Dallas, Austin, and San Antonio. PM_2.5_ levels in the states around the Great Lakes and in the Northeastern U.S. have rarely been affected by fire smoke during our study period.

Conducting large-scale epidemiological studies to investigate the impact of fire smoke on human health has been challenging, largely due to the difficulty in estimating spatially resolved exposure to fire smoke PM_2.5_. Recently, a few modeling studies of smoke PM_2.5_ concentrations in the CONUS have been conducted with spatial resolutions ranging from 10 to 15 km.^27,48^ Using machine learning models such as those presented in this study allows the integration of CTM fire simulations, high-resolution satellite remote sensing of fire smoke, and the broader spatial representation of the PurpleAir sensor network to achieve high spatial resolution (1 km), high temporal resolution (daily), and full-coverage of the CONUS for a 12 year period. The temporal trend and spatial characteristics of our model-predicted smoke PM_2.5_ concentrations align with those of major fire events across the country. For example, data from the National Interagency Fire Center^49^ showed that fire activities in Southern California, eastern Texas, and southern North Carolina and Tennessee in 2007 were 125 and 121% of previous 10 year average, respectively. The acres burned in the Rocky Mountains were 367 and 351% of previous 10 year average in 2012 and 2017, respectively, and our model successfully captures these features. Compared with uncalibrated CMAQ simulations of smoke PM_2.5_ (Figure S5, panel A), our predictions better represent the spatial and temporal distribution of fire smoke. For instance, our model captured the high smoke PM_2.5_ values in the West and Southeast during the extreme fire years, such as 2007, 2017, and 2018 (Figure 2), and low smoke PM_2.5_ values in 2015, which have same temporal trend as reported by National Interagency Fire Center.^49^ In addition, our model was able to capture finer spatial features due to its high spatial resolution at 1 km. Compared with previous smoke PM_2.5_ estimations with coarse resolution, our predictions provided a clearer boundary of the smoke-impacted areas and captured the detailed variability of population exposure levels. As illustrated in Figure S6, population within an area of 100 km^2^ in Sacramento, California, were able to be assigned to 100 unique smoke PM_2.5_ values based on their locations rather than one average value, which offers the feasibility for high-resolution health impact studies.

To the best of our knowledge, our study is the first large-scale attempt to use calibrated PM_2.5_ concentration measurements from low-cost sensors such as PurpleAir monitors in conjunction with AQS monitors to better characterize the spatial variability of smoke PM_2.5_. Previous research has shown that low-cost sensor measurements can increase the likelihood of detecting wildfire smoke,^19,31^ and integrating low-cost sensor data with regulatory measurements has allowed for better training of satellite-based machine learning models for identifying air pollution hotspots.^24,50^ In our study, PurpleAir sensors reported extreme PM_2.5_ concentrations over 200 μg/m^3^ during the Camp fire in California, while the highest AQS measurement was approximately 100 μg/m^3^ as there were no AQS monitors located near the smoke plumes. Including the high PM_2.5_ measurements from PurpleAir in our training data set reduced the model underestimation on high PM_2.5_ values. For instance, the smoke PM_2.5_ prediction from models without PurpleAir (Figure S5, panel B) was biased low in California where high smoke PM_2.5_ values always occurred and the difference of annual smoke PM_2.5_ predictions between models with and without PurpleAir measurements reached up to 16 μg/m^3^ in 2018. While PurpleAir measurements are not available prior to 2016, our models were developed using data from 2007 to 2018. Since our models do not incorporate time indicators, the influence of PurpleAir data extends across the entire study period rather than being limited to the years when PurpleAir measurements were available. Unlike earlier studies which attributed the deviation from background levels of PM_2.5_ to smoke using ground total PM_2.5_ measurements, satellite-based smoke plume identification, and air trajectories,^27,48^ we employed two different CMAQ simulations, with and without fire emissions, along with satellite-based HMS smoke contours to more accurately label smoke-impacted areas and days. Our approach facilitates independent modeling of both background PM_2.5_ and total PM_2.5_ accounting for smoke impact nationwide.

### Effect of Fire Smoke on National PM_2.5_ Concentration Levels

**Figure 3:**
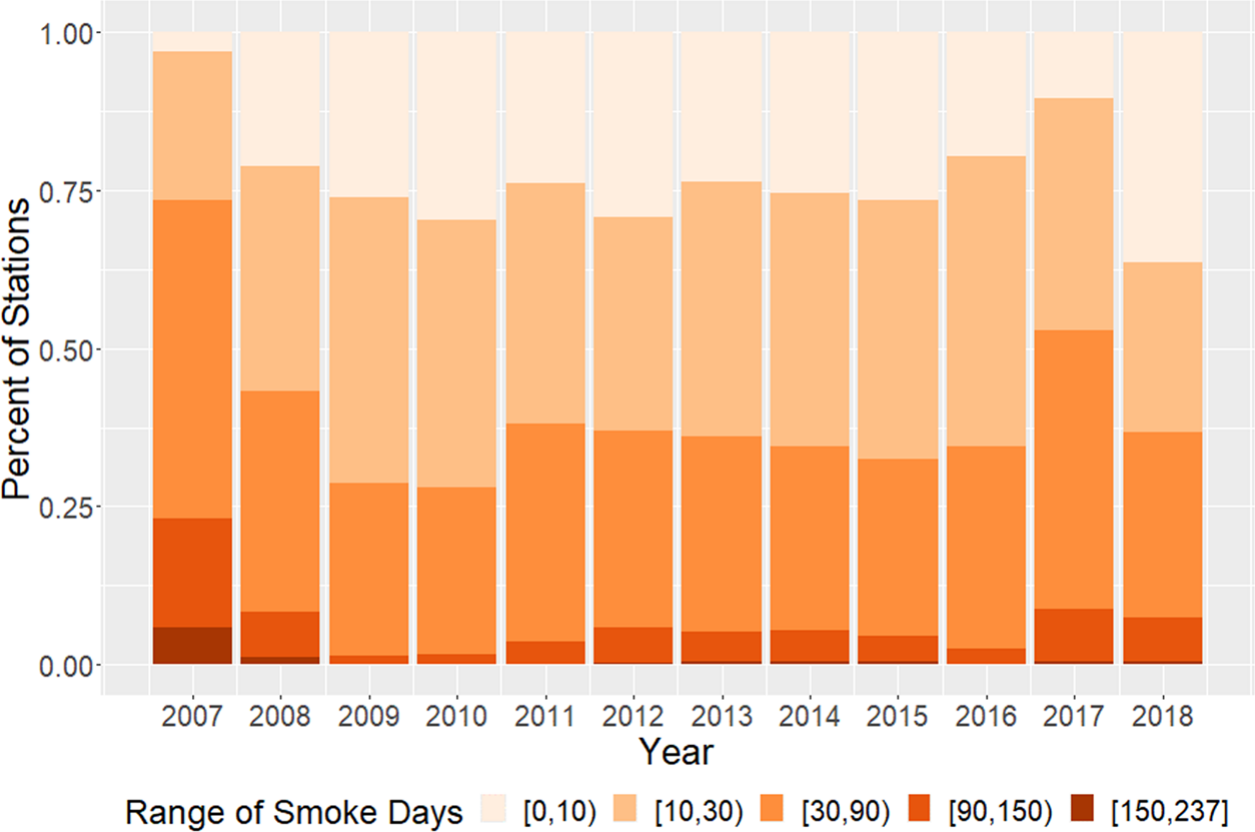
Fractions of EPA PM_2.5_ monitoring locations significantly affected by fire smoke from 2007 to 2018.

Using our daily model predictions, we assessed the impact of fire smoke on the regulatory air quality monitoring network. We defined a smoke impact day as when fire smoke contributed more than 25% of the model-estimated daily total PM_2.5_ mass concentration at the location of an air quality monitoring station included in the EPA AQS. Around 40% of the 1836 AQS monitoring sites have experienced smoke impact days for more than a month each year during our study period (Figure 3). In 2009 and 2010 when our model predicted the lowest smoke impact on national PM_2.5_ levels, over 25% of the national ambient PM_2.5_ monitoring network was under a significant smoke impact for more than a month. In intensive fire years such as 2017, 50% of all monitoring locations were affected for at least a month, indicating a widespread impact at the national scale. During the worst fire year of 2007, 25% of all monitoring locations were affected for more than 90 days. Smoke impact on air quality was highest in summer and fall in most years. However, in low fire years such as 2009 and 2010, fire smoke had the greatest impact in spring and fall.

### AQS’s Representativeness of Population Exposure to Fire Smoke

Using our model predictions and annual population estimates at a 1 km resolution, we estimated the U.S. population affected by fire smoke. As shown in Table 1, nearly the entire population in the CONUS, ranging from 95% in 2018 to 100% in 2007, has been exposed to significant fire smoke PM_2.5_, defined as over 25% smoke contribution to total PM_2.5_, for at least 1 day per year. On average, a slightly higher percentage of people living outside the vicinity of an EPA AQS monitoring station (defined by a 5 km radius) have been exposed to fire smoke. The average duration of population exposure to fire smoke showed a more substantial difference. On average, people living outside the vicinity of an AQS monitoring station experienced 25 smoke impact days, 34% (ranging from −8% in 2018 to 70% in 2012) greater than people living near an AQS station. While the mean model-estimated total PM_2.5_ concentration in regions near an AQS station (10.79 μg/m^3^) is 22% greater than that in regions without AQS coverage (8.87 μg/m^3^), the estimated smoke PM_2.5_ concentration shows the opposite trend, with a 30% decrease (0.50 vs 0.65 μg/m^3^). Since the majority of AQS stations are located in urban areas, these findings suggest that using EPA observations alone may substantially underestimate both the duration and the concentration of the fire smoke exposure of the rural and suburban population. Figures based on Table 1 are shown in the Supporting Information (Figure S7) to make the temporal trend visible.

**Table 1:** Fire Smoke Impact on the U.S. Population

year	total population (population without AQS coverage) (million)	smoke-impacted total population (smoke-impacted population without AQS coverage) (million)	smoke impact days among population with AQS coverage (among population without AQS coverage)	total PM_2.5_ (smoke PM_2.5_) with AQS coverage	total PM_2.5_ (smoke PM_2.5_) without AQS coverage
2007	300.1 (73.6)	299.7 (73.6)	38.2 (54.6)	11.90 (0.96)	9.87 (1.11)
2008	302.6 (74.1)	298.3 (72.6)	21.2 (22.4)	10.42 (0.32)	8.26 (0.38)
2009	305.5 (70.9)	300.3 (69.9)	13.5 (19.4)	11.20 (0.25)	8.45 (0.22)
2010	307.0 (72.0)	285.7 (71.6)	12.8 (22.6)	10.83 (0.57)	9.73 (0.77)
2011	310.0 (72.9)	307.9 (72.7)	16.4 (25.7)	11.43 (0.51)	9.14 (0.73)
2012	299.9(72.0)	289.0 (71.6)	11.9 (20.3)	10.35 (0.53)	9.28 (0.83)
2013	313.1 (74.0)	308.0 (72.9)	16.7 (19.1)	11.57 (0.61)	9.34 (0.66)
2014	317.3 (74.6)	310.8 (74.3)	16.9 (22.5)	9.40 (0.31)	8.74 (0.40)
2015	319.8 (74.9)	313.2 (74.6)	14.4 (19.2)	9.37 (0.48)	7.91 (0.64)
2016	321.5 (74.9)	319.7 (74.9)	17.6 (25.0)	9.30 (0.31)	7.98 (0.48)
2017	324.1 (74.6)	321.6 (74.5)	26.2 (33.8)	11.22 (0.97)	8.78 (0.92)
2018	325.6 (74.8)	308.9 (73.1)	20.2 (18.5)	10.51 (0.61)	8.95 (0.65)
average	312.2 (73.6)	305.3 (73.0)	18.8 (25.2)	10.79 (0.50)	8.87 (0.65)

### Impact of Fire Smoke on Attainment Status with the Proposed New PM_2.5_ Standard

In January 2023, the U.S. EPA proposed to lower the NAAQS for annual mean PM_2.5_ concentrations, calculated as the average of the past three years, to a value between 9 and 10 μg/m^3^. We estimated the total population as well as the number of AQS monitoring sites that would reside in nonattainment areas under the new standard (Tables S4 and S5). Without considering the impact of fire smoke, an average of 116.83 million people (from 68.73 million in 2016 to 148.74 million in 2013) and 30% of all AQS monitoring sites (from 15% in 2017 to 40% in 2011) in the CONUS would be in areas with annual mean PM_2.5_ concentrations equal to or above 10 μg/m^3^. When we considered the fire smoke contribution to PM_2.5_ levels, an additional 21.4 million people and 6% of AQS monitors would reside in nonattainment areas. Under the stricter standard of 9 μg/m^3^, the average affected population would increase to 167.23 million without considering the effect of fire smoke and 197.68 million (ranging from 153.73 million in 2016 to 225.27 million in 2013) with the contribution of fire smoke. Regarding air quality monitoring, an average of 41% of all AQS monitoring sites would fall into nonattainment areas. When the contribution of fire smoke was considered, this percentage rose to 50% (ranging from 37% in 2016 to 58% in 2011 and 2012).

As the increasing regulation of emissions of PM_2.5_ and its precursors from anthropogenic sources have effectively improved air quality in most parts of the U.S., fire emissions are becoming a major contributor of PM_2.5_. The proximity of large populations to wildland fires poses a nontrivial threat to public health and compliance with ambient air quality standards. According to EPA,^51^ approximately 20.9 million Americans (2010 population) reside in PM_2.5_ nonattainment areas based on the current NAAQS as of 2023. This number changed to around 21.7 million, based on our model-estimated county-level annual total PM_2.5_ in 2018 and current NAAQS of 12 μg/m^3^. Our model estimated that 95.9–146.3 million more people would live in nonattainment areas if the annual mean PM_2.5_ NAAQS were lowered to between 9 and 10 μg/m^3^. Our calculations also suggested that taking the impact of fire smoke into account would result in an additional 21.4–30.5 million people falling into nonattainment areas. As most wildland fires start in rural areas, fire smoke PM_2.5_ would disproportionally affect suburban and rural populations. The comprehensive spatial coverage of our model estimates would enable future research on the differential health effects of air pollution exposure associated with altered PM_2.5_ composition in these communities.

## Supporting Information Available

Predictors in statistical modeling; Figures S1–S7; Tables S1–S5; and references (PDF)
