# Supplementary material for: Wildland Fires Worsened Population Exposure to PM2.5 Pollution in the Contiguous United States
Source: Environ Sci Technol. 2023 Nov 9;57(48):19990–8. doi: 10.1021/acs.est.3c05143 (PMC10702504; doi:10.1021/acs.est.3c05143)
Supplement: Supplementary file 1 — es3c05143_si_001.pdf [file es3c05143_si_001.pdf]

Supporting Information for "Wildland fires worsens population exposure to $PM_{2.5}$ pollution in the Contiguous United States"

Danlu Zhang[1], Wenhao Wang[1], Yuzhi Xi[1], Jianzhao Bi[2], Yun Hang[1], Qingyang Zhu[1], Qiang Pu[1], Howard Chang[3], Yang Liu[1]*

[1] Gangarosa Department of Environmental Health, Rollins School of Public Health, Emory University; Atlanta, GA, United States

[2] Department of Environmental & Occupational Health Sciences, School of Public Health, University of Washington; Seattle, WA, United States

[3] Department of Biostatistics and Bioinformatics, Rollins School of Public Health, Emory University; Atlanta, GA, United States

*Email: yang.liu@emory.edu

Contents: 21 pages, supporting text, Figures S1 to S7, Tables S1 to S5 and supporting information references

Supporting Information Text

Materials and Methods

$PM_{2.5}$ Predictor Variables for Statistical Modeling

To develop the $PM_{2.5}$ prediction models, we collected a large array of predictor variables, including satellite-retrieved aerosol, cloud, and smoke plumes information, gridded meteorology, population, land cover and topographic data.

The Multi-Angle Implementation of Atmospheric Correction (MAIAC) is an algorithm using time series analysis and a combination of pixel and image-based processing based on the Moderate Resolution Imaging Spectroradiometer (MODIS) Collection 6 measurements to improve the aerosol retrieval accuracy and optimize the spatial resolution to 1 km [1]. MAIAC AOD at 550 nm from the Terra (overpass at 10:30 a.m. local time) and Aqua (overpass at 1:30 p.m. local time) were obtained from NASA EOSDIS Land Processes Distributed Active Archive Center [2]. Cloud cover and water or ice glint reflectance can cause a considerable proportion of random missing data in satellite AOD [3,

[4], so we extracted modelled AOD data from Monitoring Atmospheric Composition and Climate - Interim Implementation (MACC-II) project, processed by Copernicus Atmosphere Monitoring Service (CAMS). CAMS AOD at five different wavelengths (469 nm, 550 nm, 670 nm, 865 nm and 1240 nm) were downloaded from the European Centre for Medium-Range Weather Forecasts (ECMWF) with a spatial resolution of $0.75^{\circ} \times 0.75^{\circ}$ and a temporal resolution of 3 hours [5].

Cloud fractions at a 5 km resolution were obtained from Moderate Resolution Imaging Spectroradiometer (MODIS) Level-2 Cloud product [6]. Remotely sensed infrared visible and near infrared solar reflected radiances were used to derive the cloud fraction [7]. It has been indicated that MODIS cloud product had an agreement with the lidar about 85% of the time [8]. Only daytime cloud fraction was extracted to represent the cloud condition at satellite overpass times. Smoke plumes were taken from the National Oceanic and Atmospheric Administration/National Environmental Satellite, Data, and Information Service (NOAA/NESDIS) Satellite Analysis Branch's Hazard Mapping System (HMS), based on visual classification using GOES-16 and GOES-17 ABI true-color imagery [9].

Attributes of smoke plumes included the plume density along with start and end times of the satellite image sequence used to outline the smoke polygon. Since one grid cell can overlap with multiple smoke plumes in each day, we calculated daily weighted average plume density for each grid cell. In this calculation, the weight is smoke plume duration considered as the difference between start and end times.

Gridded hourly meteorological factors, including air temperature, specific humidity, surface pressure, surface downward longwave and shortwave radiation, U and V wind component, total precipitation, and potential evaporation, with a spatial resolution of $0.125^\circ \times 0.125^\circ$, were obtained from the North American Land Data Assimilation System phase 2 (NLDAS-2) [10]. Planetary boundary layer height was downloaded from the North American Regional Reanalysis (NARR) at a spatial resolution of 32 km [11]. Daily averaged meteorological factors were calculated between 9 a.m. and 3 p.m. at local time zones to represent the weather condition at satellite overpass times. The land cover data at 30-meter resolution were obtained from the National Land Cover Database (NLCD) [12]. NLCD released land cover for years of 2008, 2011, 2013, 2016 and 2019. Land cover data in gap

years were taken from later released products. The percentage of each land cover type, such as water, developed area, barren, forest, shrubland, herbaceous area, cultivated area, and wetland, was calculated by reclassifying 20 original land cover types. Annual population counts at 30 arc second spatial resolution were download from Oak Ridge National Laboratory's LandScan [13]. Road network data, including road types of highways, primary road, secondary road, tertiary road, and local road, at a 5 arcminutes resolution were downloaded from the Global Roads Inventory Project (GRIP) [14]. The elevation data was extracted from the Terra Advanced Spaceborne Thermal Emission and Reflection Radiometer (ASTER) Global Digital Elevation Model (GDEM) Version 3 (ASTGTM) at a resolution of 1 arc second [15].

Satellite AOD Gap-filling Approach

To address the missing data issue of satellite AOD, we customized a two-step approach to improve the spatial coverage of satellite AOD data. First, we fitted linear regression

models between daily Terra and Aqua AOD to predict missing Terra AOD when only Aqua AOD was available in those grids and vice versa. Daily averaged AOD in each grid was calculated as the mean from both satellites. Second, we developed a RF-based model to predict the daily AOD over grids where both Terra and Aqua AOD were missing [3, 4]. The equation is expressed as

$$AOD_{(s,t)} = f(cloud\ fraction_{(s,t)}, air\ temperature_{(s,t)}, specific\ humidity_{(s,t)},$$

$$precipitation_{(s,t-1)}, elevation_{(s,t)}, \sum_{i=1}^{M}\sum_{j=1}^{N} CAMS\ AOD_{(s,t)})$$

where s is the location of a grid cell, and t is the time of an observation. M is the number of wavelength (M = 5) and N represents the available hours (N = 8). MAIAC AOD was the dependent variable. The independent variables consisted of cloud fraction, air temperature, specific humidity, precipitation on the previous day, elevation, and separate CAMS AOD at five wavelengths and available hours. To balance the computing time and prediction accuracy, we kept the training dataset in each single day no more than 750,000, which indicates if the full training data was over 750,000, we randomly selected 750,000

observations to train the RF-based gap-filling model and *num.tree* and *mtry* were set as 50 and 10, respectively [4].

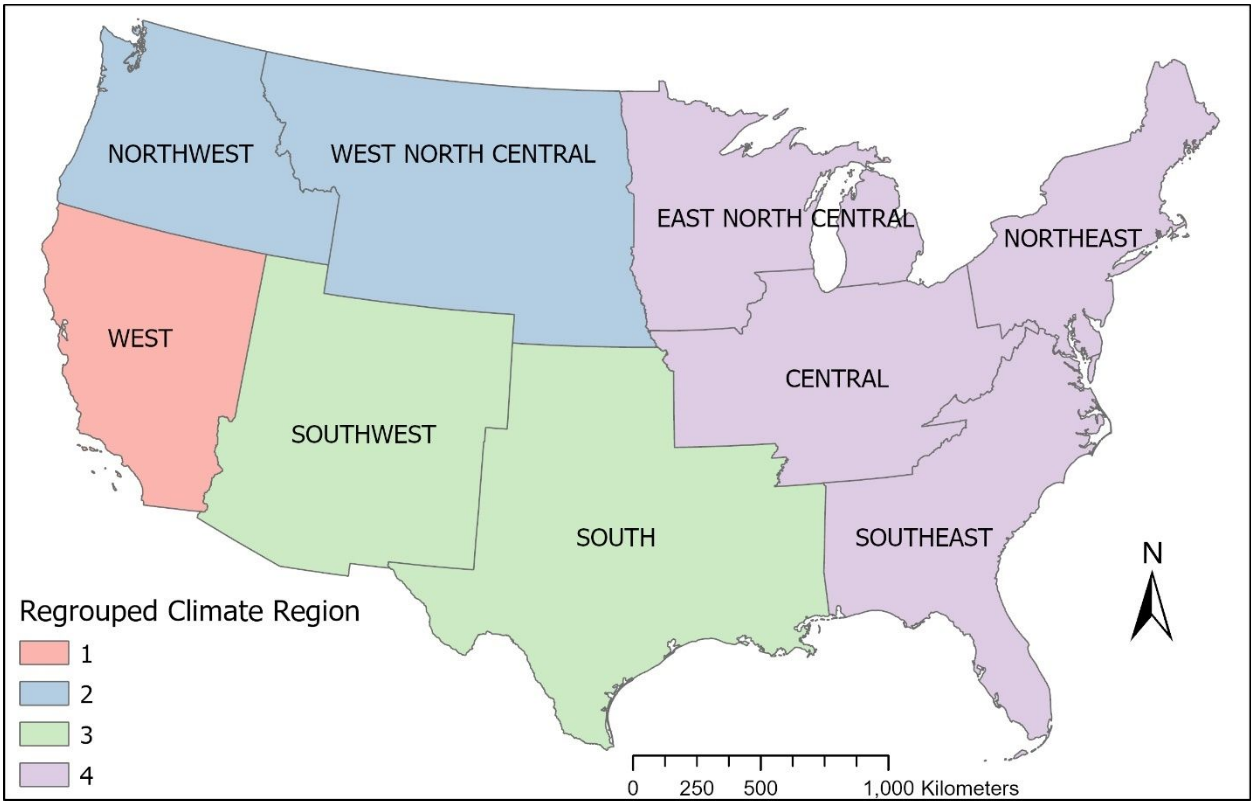

Figure S1. Regrouped nine climate regions in four parts.

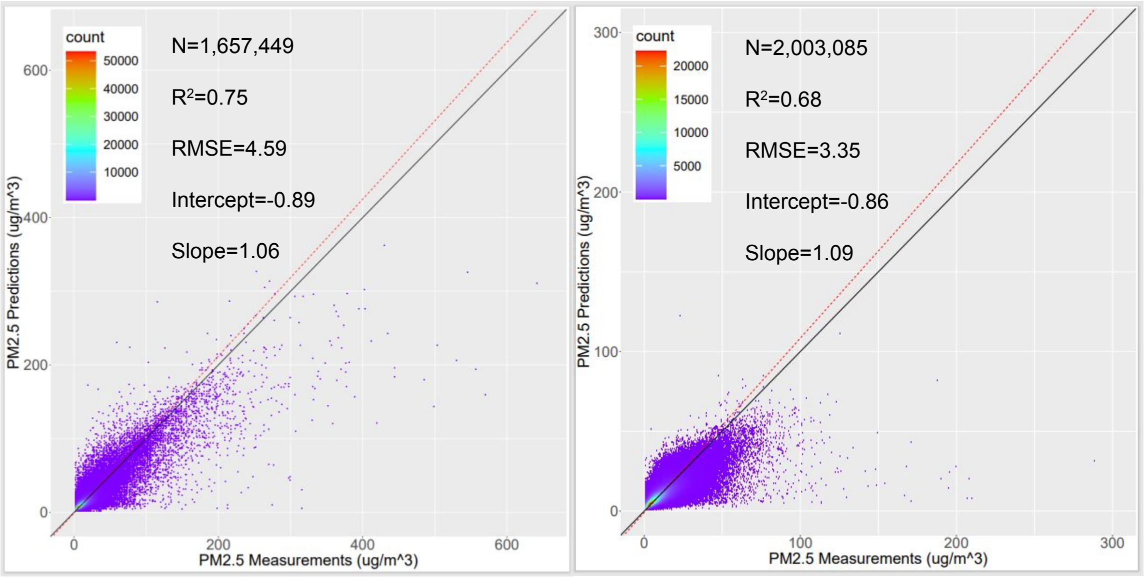

Figure S2. Scatter Plot for 20-fold Cross-validation of Daily $PM_{2.5}$ in fire (left) and non-fire (right) models. Red dashed line is the regression line. Black line is the 1:1 line.

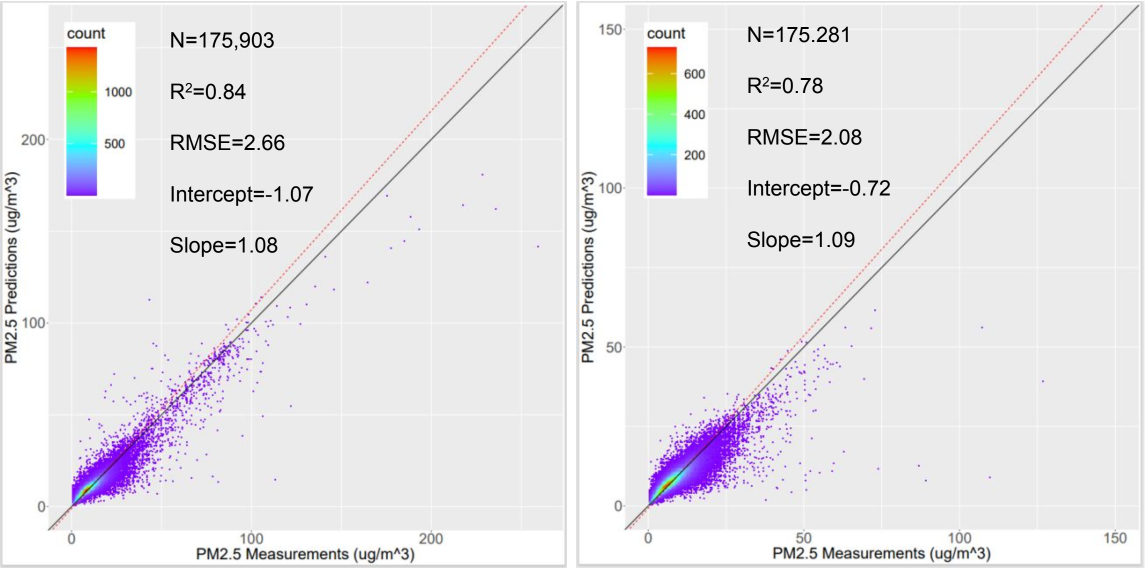

Figure S3. Scatter Plot for 20-fold Cross-validation of Monthly $PM_{2.5}$ in fire (left) and non-fire (right) models. Red dashed line is the regression line. Black line is the 1:1 line.

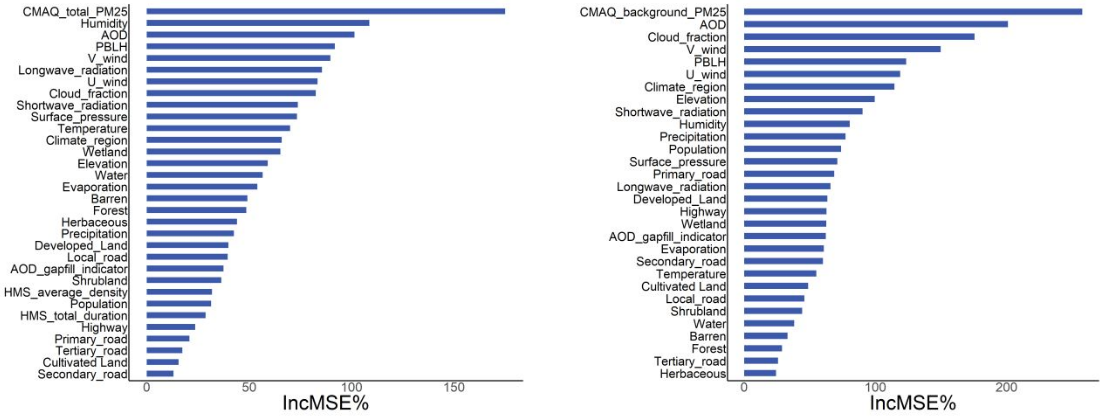

Figure S4. Variables importance rank of fire (left) and non-fire (right) models.

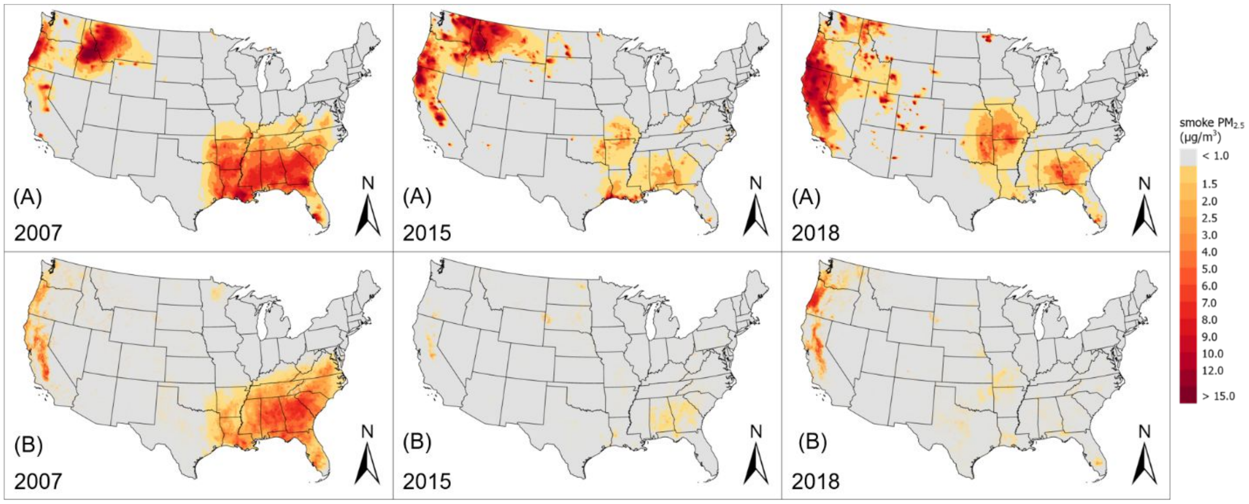

Figure S5. (Panel A) Interpolated CMAQ simulations of smoke $PM_{2.5}$ in 2007, 2015 and 2018. (Panel B) Annual smoke $PM_{2.5}$ predictions in 2007, 2015 and 2018 from models without PurpleAir measurements.

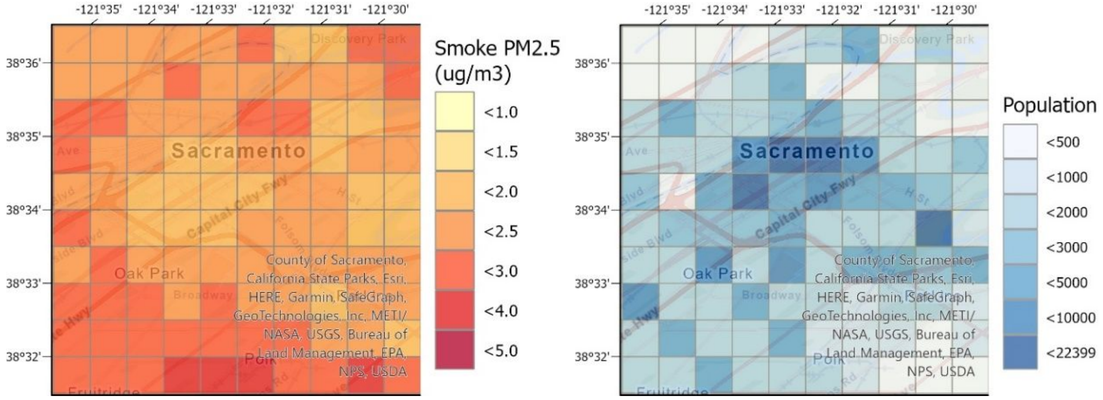

Figure S6. 2018 annual level smoke $PM_{2.5}$ prediction distributions in the 100 km^2 area in Sacramento, California (left). Population count at 1 km resolution in the same area (right).

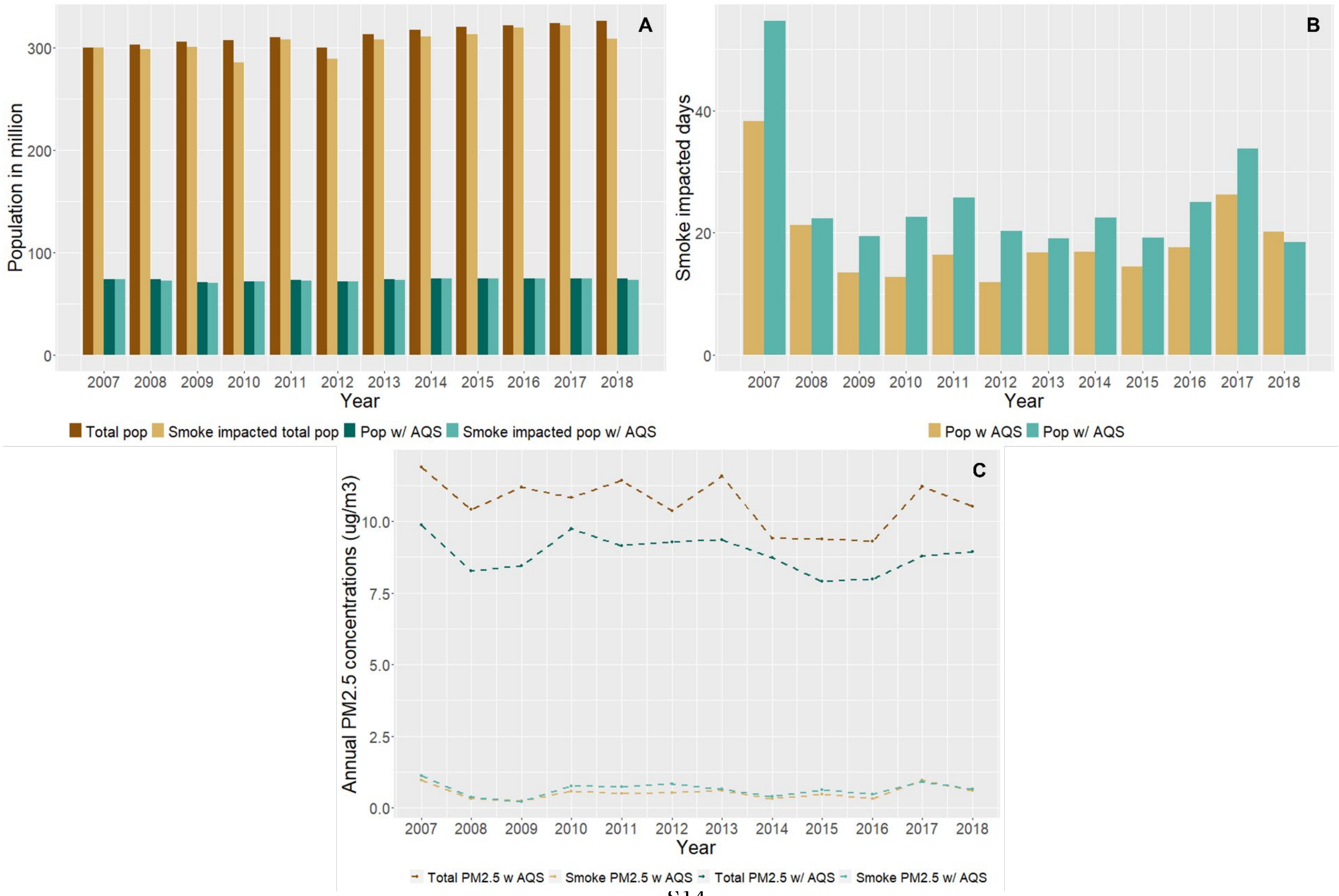
A
Population in million
300
200
100
0
2007 2008 2009 2010 2011 2012 2013 2014 2015 2016 2017 2018
Year
Total pop
Smoke impacted total pop
Pop w/ AQS
Smoke impacted pop w/ AQS
B
Smoke impacted days
40
20
0
2007 2008 2009 2010 2011 2012 2013 2014 2015 2016 2017 2018
Year
Pop w AQS
Pop w/ AQS
C
Annual PM2.5 concentrations (ug/m3)
10.0
7.5
5.0
2.5
0.0
2007 2008 2009 2010 2011 2012 2013 2014 2015 2016 2017 2018
Year
Total PM2.5 w AQS
Smoke PM2.5 w AQS
Total PM2.5 w/ AQS
Smoke PM2.5 w/ AQS

Figure S7. A. Annual total population (total pop), smoke impacted total population (smoke impacted total pop), population among areas without AQS coverage (pop w/ AQS) and smoke impacted population among areas without AQS coverage (smoke impacted pop w/ AQS). **B**. Smoke impacted days among population with AQS coverage (pop w AQS) and smoke impacted days among population without AQS coverage (pop w/ AQS). **C**. Annual total $PM_{2.5}$ and smoke $PM_{2.5}$ concentrations with AQS coverage (total $PM_{2.5}$ w AQS and smoke $PM_{2.5}$ w AQS) and annual total $PM_{2.5}$ and smoke $PM_{2.5}$ concentrations without AQS coverage (total $PM_{2.5}$ w/ AQS and smoke $PM_{2.5}$ w/ AQS

Table S1. Details of CMAQ model versions, emission, and chemistry information.

Year	NEI year	CMAQ version	BEIS version	EGU CEM data	Gas phase chemistry	PM chemistry	Boundary inflow	WRF version	Primary organic aerosol semivolatile?
2007	2008 NEI	v5.0.1	3.14	2007	CB05	AERO6	GEOS-CHEM	v3.4	N
2008	2008 NEI	v5.0.1	3.14	2008	CB05	AERO6	GEOS-CHEM	v3.4	N
2009	2008 NEI	v5.0.1	3.14	2009	CB05	AERO6	GEOS-CHEM	v3.4	N
2010	2008 NEI	v5.0.1	3.14	2010	CB05	AERO6	GEOS-CHEM	v3.4	N
2011	2011 NEI	v5.0.1	3.14	2011	CB05	AERO6	GEOS-CHEM	v3.4	N
2012	2011 NEI	v5.0.2	3.14	2012	CB05	AERO6	GEOS-CHEM	v3.4	N
2013	2011NEIv2	v5.2	3.6.1	2013	CB6r3	AERO6	GEOS-CHEM	v3.8	N
2014	2014NEIv1	v5.2	3.6.1	2014	CB6r3	AERO6	GEOS-CHEM	v3.8.1	N
2015	2014NEIv2	v5.2.1	3.6.1	2015	CB6r3	AERO6	Hemispheric CMAQ	v3.8.1	N
2016	2014NEIv2	v5.2.1	3.6.1	2016	CB6r3	AERO6	Hemispheric CMAQ	v3.8.1	N
2017	2014NEIv2	v5.2.1	3.6.1	2017	CB6r3	AERO6	Hemispheric CMAQ	v3.8.1	N

2018	2014NEIv2	v5.3	3.6.1	2018	CB6r3	AERO7	Hemispheric CMAQ	v3.8.1	Y

Table S2. Variables Used in Random Forest Models

Ground Monitors	EPA $PM_{2.5}$ observations, Type: continues, Unit: μg/m^3
	PurpleAir $PM_{2.5}$ observations, Type: continues, Unit: μg/m^3
CMAQ Simulations	Total $PM_{2.5}$ simulations, Type: continues, Unit: μg/m^3
	Background $PM_{2.5}$ simulations, Type: continues, Unit: μg/m^3
MAIAC AOD	Gap-filled AOD, Type: continues, Unit: dimensionless
	Gap-filling indictor, Type: categorical, 0 = observed, 1 = gap-filled
Cloud fractions	Cloud fraction, Type: continues, Unit: dimensionless
Meteorological factors	Air temperature, Type: continues, Unit: K
	Specific humidity, Type: continues, Unit: kg/kg
	Surface pressure, Type: continues, Unit: Pa
	Surface downward longwave radiation, Type: continues, Unit: W/m^2
	Surface downward shortwave radiation, Type: continues, Unit: W/m^2
	U wind component, Type: continues, Unit: m/s
	V wind component, Type: continues, Unit: m/s
	Total precipitation, Type: continues, Unit: dimensionless
	Potential evaporation, Type: continues, Unit: kg/m^2

	Planetary boundary layer height, Type: continues, Unit: m
HMS data	Total duration time, Type: continues, Unit: minute
	Average smoke density, Type: continues, Unit: mg/m^3
Land use data	Water fraction, Type: continues, Unit: dimensionless
	Developed fraction, Type: continues, Unit: dimensionless
	Barren fraction, Type: continues, Unit: dimensionless
	Forest fraction, Type: continues, Unit: dimensionless
	Shrubland fraction, Type: continues, Unit: dimensionless
	Herbaceous fraction, Type: continues, Unit: dimensionless
	Cultivated fraction, Type: continues, Unit: dimensionless
	Wetland fraction, Type: continues, Unit: dimensionless
	Population, Type: continues, Unit: dimensionless
	Highway length, Type: continues, Unit: m
	Primary road length, Type: continues, Unit: m
	Secondary road length, Type: continues, Unit: m
	Tertiary road length, Type: continues, Unit: m
	Local road length, Type: continues, Unit: m
Elevation	Elevation, Type: continues, Unit: m

Climate regions	Climate region, Type: categorical, 1-Central, 2-East North Central, 3-Northeast, 4-Northwest, 5-South, 6-Southeast, 7-Southwest, 8-West, 9-West North Central

Table S3. Summaries of overall, spatial and temporal 20-fold CV for models in the smoke-impacted and no-smoke regions

**Smoke-impacted Model**		**N**	**R^2**	**RMSE**	**Intercept**	**Slope**
Daily	Random 20-fold CV	1,657,449	0.75	4.59	-0.89	1.06
	Spatial 20-fold CV	1,657,449	0.59	5.88	-1.35	1.07
	Temporal 20-fold CV	1,657,449	0.67	5.18	-0.68	1.05
Monthly	Random 20-fold CV	175,903	0.84	2.66	-1.07	1.08
	Spatial 20-fold CV	175,903	0.67	3.82	-1.89	1.12
	Temporal 20-fold CV	175,903	0.79	3.00	-1.21	1.10
**No-smoke Model**		**N**	**R^2**	**RMSE**	**Intercept**	**Slope**
Daily	Random 20-fold CV	2,003,085	0.68	3.35	-0.86	1.09
	Spatial 20-fold CV	2,003,085	0.47	4.30	-0.96	1.07

	Temporal 20-fold CV	2,003,085	0.63	3.59	-0.70	1.07
Monthly	Random 20-fold CV	175,281	0.78	2.08	-0.72	1.09
	Spatial 20-fold CV	175,281	0.54	2.98	-1.00	1.09
	Temporal 20-fold CV	175,281	0.74	2.24	-0.65	1.08

Table S4. Estimated number of AQS monitors that would fall into nonattainment areas by the proposed $PM_{2.5}$ standard (annual mean, averaged over 3 years).

Year[a]	AQS monitors w/ total $PM_{2.5} \geq 9$ μg/m^3	AQS monitors w/ background $PM_{2.5} \geq 9$ μg/m^3	AQS monitors w/ total $PM_{2.5} \geq 10$ μg/m^3	AQS monitors w/ background $PM_{2.5} \geq 10$ μg/m^3
2009	55%	46%	42%	38%
2010	54%	47%	40%	36%
2011	58%	51%	45%	40%
2012	58%	49%	44%	37%
2013	57%	48%	45%	37%
2014	53%	44%	39%	33%
2015	48%	39%	33%	27%
2016	37%	30%	20%	16%
2017	39%	28%	21%	15%
2018	45%	33%	27%	20%
Average	50%	41%	36%	30%

[a] Annual mean indicates the averages of previous three years (i.e. annual mean in 2009 is averaged over 2007-2009).

Table S5. Estimated population affected by the proposed $PM_{2.5}$ standard (annual mean, averaged over 3 years).

Year[a]	Population in nonattainment areas w/ total $PM_{2.5}$ ≥ 9 μg/m^3 (million)	Population in nonattainment areas w/ background $PM_{2.5}$ ≥9 μg/m^3 (million)	Population in nonattainment areas w/ total $PM_{2.5}$ ≥10 μg/m^3 (million)	Population in nonattainment areas w/ background $PM_{2.5}$ ≥10 μg/m^3 (million)
2009	199.01	176.73	153.45	137.41
2010	197.00	177.29	143.81	132.15
2011	215.93	191.76	164.78	144.70
2012	219.27	185.09	168.20	139.08
2013	225.27	192.81	176.82	148.74
2014	214.34	182.20	155.63	132.47
2015	197.18	166.16	132.06	109.22
2016	153.73	128.79	83.81	68.73
2017	167.62	125.18	90.91	68.83
2018	187.47	146.33	113.21	87.03
Average	197.68	167.23	138.27	116.83

[a] Annual mean indicates the averages of previous three years (i.e. annual mean in 2009 is averaged over 2007-2009).

142

SI References

1. Lyapustin, A.; Wang, Y.; Korkin, S.; Huang, D., MODIS Collection 6 MAIAC algorithm. *Atmos. Meas. Tech.* **2018,** *11* (10), 5741-5765.
2. Lyapustin, A., Wang, Y, MCD19A2 MODIS/Terra+Aqua Land Aerosol Optical Depth Daily L2G Global 1km SIN Grid V006. NASA EOSDIS Land Processes DAAC, 2018.
3. Bi, J.; Belle, J. H.; Wang, Y.; Lyapustin, A. I.; Wildani, A.; Liu, Y., Impacts of snow and cloud covers on satellite-derived PM2. 5 levels. *Remote sensing of environment* **2019,** *221*, 665-674.
4. Stafoggia, M.; Bellander, T.; Bucci, S.; Davoli, M.; de Hoogh, K.; de' Donato, F.; Gariazzo, C.; Lyapustin, A.; Michelozzi, P.; Renzi, M.; Scortichini, M.; Shtein, A.; Viegi, G.; Kloog, I.; Schwartz, J., Estimation of daily PM10 and PM2.5 concentrations in Italy, 2013–2015, using a spatiotemporal land-use random-forest model. *Environment International* **2019,** *124*, 170-179.
5. Inness, A.; Ades, M.; Agustí-Panareda, A.; Barré, J.; Benedictow, A.; Blechschmidt, A. M.; Dominguez, J. J.; Engelen, R.; Eskes, H.; Flemming, J.; Huijnen, V.; Jones, L.; Kipling, Z.; Massart, S.; Parrington, M.; Peuch, V. H.; Razinger, M.; Remy, S.; Schulz, M.; Suttie, M., The CAMS reanalysis of atmospheric composition. *Atmos. Chem. Phys.* **2019,** *19* (6), 3515-3556.
6. Platnick, S., S. A. Ackerman, M. D. King, K. Meyer, W. P. Menzel, R. E. Holz, B. A. Baum, and P. Yang, MODIS atmosphere L2 cloud product (06_L2). NASA MODIS Adaptive Processing System, Goddard Space Flight Center, 2015.
7. Platnick, S., S. Ackerman, M. King, G. Wind, K. Meyer, P. Menzel, R. Frey, R. Holz, B. Baum, and P. Yang, MODIS atmosphere L2 cloud product (06_L2). **2015**.
8. Ackerman, S.; Holz, R.; Frey, R.; Eloranta, E.; Maddux, B.; McGill, M., Cloud detection with MODIS. Part II: validation. *Journal of Atmospheric and Oceanic Technology* **2008,** *25* (7), 1073-1086.
9. Hazard Mapping System Fire and Smoke Product. https://www.ospo.noaa.gov/Products/land/hms.html.
10. NLDAS-2 Forcing Dataset Information. https://ldas.gsfc.nasa.gov/nldas/v2/forcing.
11. Fedor Mesinger, G. D., Eugenia Kalnay, Kenneth Mitchell, Perry C. Shafran, Wesley; Ebisuzaki, D. J., Jack Woollen, Eric Rogers, Ernesto H. Berbery, Michael B. Ek, Yun; Fan, R. G., Wayne Higgins, Hong Li, Ying Lin, Geoff Manikin, David Parrish, and; Shi, W., A long-term, consistent, high-resolution climate dataset for the North American domain, as a major improvement upon the earlier global reanalysis datasets in both resolution and accuracy. *the Bulletin of the American Meteorological Society* **2004**.

12. Dewitz, J., and U.S. Geological Survey, National Land Cover Database (NLCD) 2019 Products (ver. 2.0, June 2021): U.S. Geological Survey data release. 2021-06-04 ed.; 2021.

13. Rose, A., Weber, E., Moehl, J., Laverdiere, M., Yang, H., Whitehead, M., Trombley, N., Sims, K., Whitlock, C., & Bhaduri, B, LandScan USA 2018. Oak Ridge National Laboratory, 2019.

14. Meijer, J. R., Huijbregts, M.A.J., Schotten, C.G.J. and Schipper, A.M., Global patterns of current and future road infrastructure. *Environmental Research Letters* **2018,** *13-064006*.

15. NASA/METI/AIST/Japan Spacesystems and U.S./Japan ASTER Science Team, ASTER Global Digital Elevation Model V003 NASA EOSDIS Land Processes DAAC, 2019.
